# A Study of Maternal Competence in Preterm Birth Condition, during the Transition from Hospital to Home: An Early Intervention Program’s Proposal

**DOI:** 10.3390/ijerph18168670

**Published:** 2021-08-17

**Authors:** Concetta Polizzi, Giovanna Perricone, Maria Regina Morales, Sofia Burgio

**Affiliations:** 1 Italian Society of Pediatric Psychology (S.I.P.Ped.), Department of Psychology, Educational Science and Human Movement, University of Palermo, 90128 Palermo, Italy; concetta.polizzi@unipa.it (C.P.); giovanna.perricone@unipa.it (G.P.); 2 Italian Society of Pediatric Psychology (S.I.P.Ped.), Mental Health Department, ASST Sette Laghi, 21100 Varese, Italy; regina.morales@libero.it

**Keywords:** parental competence, early intervention program, preterm birth

## Abstract

The study was conducted with 104 mothers (average age 32.5 years, SD 6.1) of preterm infants (very and moderately preterm but still healthy) to monitor the perceived maternal role competence from the time of hospitalisation to post-discharge, in order to define an intervention program to support mothers during this transition. A targeted Q-Sort tool (Maternal Competence Q-Sort in preterm birth) was applied at two different times as a self-observation tool for parenting competence in neonatology. A tendency towards dysregulation of the maternal role competence was detected, mainly in terms of low self-assessment and was found to worsen during post-discharge, particularly with regard to caregiving ability. This study suggests the importance of accompanying parenting competence in preterm birth conditions, not only during hospitalisation in the Neonatal Intensive Care Unit (NICU) but also following discharge in order to promote the development of premature infants. This paper reports in the last part a specific integrated psychoeducational intervention program (psychologist and nurses), which we defined precisely in light of the suggestions offered by the study data on perceived maternal competence created with the Q-sort.

## 1. Introduction

Preterm birth represents a risk condition not only for the development of the child that can lead to dysfunctional evolutionary outcomes related to all dimensions of the child’s development [1,2,3,4], but also for parents and in particular for mothers, who experience the daily hospitalisation of the newborn. In fact, parents who experience premature birth conditions may also experience adverse outcomes (anxiety, depression, perceived stress, Post Traumatic Stress Disorder) and, mothers in particular, seem to be the most affected [5,6,7]. Early and abrupt termination of pregnancy, which often appears to severely compromise the process of building a self-image as a mother and the representations of her expected child, may induce high levels of parental distress. It affects the perceived parenting competence [8,9] by devaluating the ability to practice adequate caregiving, scaffolding, and emotional and cognitive coping [10,11]. However, such distress may dysregulate parenting competence by both negative and positive misperceptions of some abilities. This maternal uneasiness impacts on the newborn’s wellbeing [7,12,13,14].

Since stress conditions elicit a number of neurophysiological responses, including stimulation of the hypothalamic–pituitary–adrenal axis (HPA), an increase in maternal cortisol levels (as the physiological response to stressful conditions) seems to predict the newborn’s negative reactivity, besides influencing his/her disposition [15]. Stress conditions may also weaken the immune defences of pregnant women and, as a consequence, of the child [16]. Mothers of preterm babies, therefore, may perceive themselves as being dysregulated in coping with daily childcare needs, in addition to being poorly equipped to recognise their child’s evolutionary demands [17,18,19,20]. Furthermore, evolutionary frailties in preterm infants may impair their ability to express emotional states, including crying, making it difficult for mothers to attune to their child’s needs. Several studies have associated preterm birth with altered maternal behaviours [21,22,23,24,25,26]. Mothers of preterm infants are likely to touch, smile at, and speak to their babies much less frequently than mothers of full-term babies [22,27]. They mention a possible correlation between prematurity and atypical parenting behaviour [28,29,30].

### The Transition from Hospital to Home for Mothers of Preterm Infants

Discharge and homecoming may become a stressful experience in mothers of preterm infants, coupled with hospitalisation in the Neonatal Intensive Care Unit (NICU), undermining their self-perception as competent parents; discharge, as period of transition to home, is a very critical period for parents, particularly for mothers who experience most of the stages of hospitalisation of the baby in NICU. In the home setting they must take on all of the caregiving responsibilities and activities and many things can go wrong if they have not been prepared for caring for an infant at home [31,32]. When premature infants are hospitalised, misperception of the self may lead mothers to ask continually for help from the medical team, for the childcare. Following discharge, the eagerly awaited homecoming may become a source of further distress, if mothers have not been adequately prepared for their return home [31,33]. Mothers who perceive their child as being in poor health and in need of constant care [34] may appear extremely concerned about recognising the child’s evolutionary problems and resources. As a result, they may feel unequipped to manage the day-to-day childcare at home without any help and also uncertain about balanced management of parental functions [35,36].

This leads to the hypothesis that, as already highlighted by literature in the field [28,30,37,38], the more extreme the preterm birth, the more mothers will struggle to interact perceptively with their child. The child will be perceived and described as unresponsive and little involved in mother–child interaction, with negative emotional states. In addition, a number of scientific studies report instances of scant vocalisations as a result of long-term NICU stay and prolonged intubation, altering the emission of sounds [39,40].

Thus, homecoming may prompt a further stress condition which is likely to alter the maternal mind-mindedness [41] and weaken the ability to understand the child’s development. According to Pediatric Psychology [11,42,43], such parental fragility may shape dysregulated mother–child relational patterns which, in turn, affect the child’s evolutionary trajectory; inadequate early care thus undermining his/her evolutionary energy [11].

Therefore, providing support and accompaniment to mothers in the transition from NICU to home is of fundamental importance [31,44,45,46,47,48,49,50,51,52,53,54,55,56].

This includes identifying possible procedures and practices to enable the mother to take charge of the preterm-birth paediatric conditions, and to integrate these procedures into the daily operations of Neonatology departments, NICUs and clinics responsible for follow-up care. Most early intervention programs in the literature [57,58,59,60,61] focus on counselling, or narrative writing techniques, or programs that are aimed at supporting developmental dimensions such as those that improve cognitive development, children’s emerging motor control and exploratory behaviours, or programs aimed at strengthening parental responsiveness in managing the stress experienced by the child; all these programs probably have an impact on the level of parenting competence, but none of these focus on parenting competence and in particular intervening simultaneously on emotional and cognitive coping, scaffolding and caregiving.

Certainly, the involvement of fathers is increasingly frequent in the research of the field both because they share with mothers the difficulties of the preterm birth condition with the consequent hospitalisation [30,62,63,64,65,66,67], and above all because they can be the main source of support for mothers [68,69].

However, in this study, as well as in the support intervention program, since our interest was focused on the complexity of the transition from hospital to home, we want to focus above all on mothers; the fathers are also significantly involved in early intervention program.

The focus on mothers is however linked, above all to the investigation tool on parental competence used (Q-sort) which is currently validated only for mothers and there are no mirror tools for fathers.

Furthermore, it is recalled that while fathers with the child’s discharge and the return to home, can return to the routine of familiar and working routine, thanks to overcoming fears for the child’s survival, mothers often find themselves alone, catapulted into a daily life that can worry them about what concerns the care of the child (will I understand what the child wants? He is still so small).

The study, therefore, in line with research on early intervention programs with parents of preterm births, in light of parental competence data made with the Q-sort methodology, has defined an integrated psychoeducational path for mothers of preterm babies.

The paper, in fact, develops along two strictly connected directions: the first relates to the monitoring of perceived maternal competence, and the second, refers to the definition and presentation of an early intervention program to support the mothers of preterm babies in the transition from hospital to home; the development of the first direction was clearly preparatory to the development of the second direction

## 2. Materials and Methods

### 2.1. Objectives and Hypothesis

As regards the first direction of this work, we refer to a study aimed at monitoring the perceived maternal role competence of premature infants’ mothers, through two measurements: during child’s hospitalisation in a Neonatal Intensive Care Unit (NICU) (T1) and in the time of immediate post-discharge (T2). It will be considered the possible effects of specific structural variables such as the severity degree of the preterm birth (severe and moderate), the type of delivery (natural or caesarean), the nature of pregnancy (natural or Assisted Reproductive Technology path), the ratio of primiparae to multiparae, and the sociocultural level and marital status. Considering the aim, the research goals are:

Goal 1: to explore maternal competence perceived in mothers of premature infants at two different times: hospitalisation in the NICU and return home after discharge. As premature birth and hospitalisation in the NICU represent a risk condition for parents, especially for mothers, we hypothesised to find a perceived maternal competence characterised by a certain degree of dysregulation in T1. Furthermore, we hypothesised that the scores on the factors of maternal competence measured with the Q-Sort may undergo a weakening on the return home (T2) compared to the time of hospitalisation (T1), especially as regards the caregiving function; in fact, the return home can represent a new stressful condition for the mother due to the absence of containment and guidance from the NICU team (neonatologists, nurses, psychologists).

Goal 2: to explore at T1 and T2, the effects of specific independent variables (degree of prematurity of child’s preterm birth, type of childbirth, nature of pregnancy, and mother’s sociocultural level, civil status, and index of parity) on the self-representation of mothers as parents.

As regards the direction of our work involved in the intervention, the following objective should be indicated:

Goal 3: define and present an early intervention program to support maternal competence in the transition from hospital to home; a program defined in the light of the suggestions offered by the study data.

### 2.2. Sample

In total, 104 mothers with premature infants hospitalised at the Neonatal Intensive Care Units (NICU) of two Italian hospitals participated in the study. Inclusion criteria were gestational age of preterm infants <34 weeks, no major brain lesions, no neurosensory deficits, no genetic syndromes and/or major malformations, and no postnatal pathologies (i.e., bronchopulmonary dysplasia, necrotising enterocolitis, retinopathy, laryngopathy).

### 2.3. Measures

The tool used to evaluate perceived maternal role competence during the two timeframes (T1 and T2) was a Q-Sort on maternal role competence in neonatology (Maternal Competence Q-sort in preterm birth), developed in compliance with procedures related to its methodology [70,71] and validated [10]. With regard to the psychometric characteristics, the tool shows a good internal subscale consistency (α = 0.89). In addition, the test–retest reliability has been reported to be appropriate (r > 0.80 and split-half method: 0.52).

The Q-sort is divided into 90 items that are related to the four factors identified by the model of parental role competence in neonatology adopted by the study [10]. The four factors are the following:

Emotional coping (A factor), intended as the ability of the mother to self-regulate and recognise her needs and emotions in managing her child’s development. The indicators of this factor are “emotional self-regulation” (A1), intended as awareness of an individual’s emotions and control of negative emotions; “decrease in emotional tension” (A2) in terms of recognition and representation of a person’s needs and the search for a response to those needs.

Cognitive coping (B factor), intended as the mother’s ability to reshape the preterm birth event cognitively in terms of planning and challenges; indicators of this are “cognitive reshaping of the preterm birth event” (B1), in terms of reshaping the representations of preterm birth as an evolutionary challenge and of the mother–child relationship through future planning; “assessment of the problems related to preterm birth and hospitalisation in the NICU” (B2), as the acknowledgement of the challenges related to the child’s hospitalisation and of the resources to deal with those challenges.

Scaffolding (C Factor) [72] defined as the mother’s ability to support her child emotionally and cognitively. In particular, in terms of “emotional scaffolding” (C1), the emotional availability or inclination to show positive experiences to the child, and “attunement” [73] in terms of emotional monitoring; “cognitive scaffolding” (C2), the mother’s ability to activate a framing process with her child by sharing attention and building routines; “relational scaffolding” (C3), the mother’s ability to interact face to face with her child and foster her child’s contact with the outside world.

Caregiving (D Factor) [74], intended as the factor that defines the mother’s ability to adjust herself to her child’s evolutionary signals and be a responsive parent. It refers to “responsiveness” (D1), the emotional and cognitive attunement to the child’s needs and the activation of adequate care; “flexible behavioural self-adjustment to the child’s needs” (D2), in terms of the ability to respond to the child’s signals and evolutionary needs, as well as to modify an individual’s own behaviour in relation to such signals, needs and context.

### 2.4. Administration Times of the Tool

First administration (T1): during hospitalisation in the NICU and, more specifically, during the 4th week of hospitalisation for Low Birth Weight (LBW) babies and between the 5th and 6th week for Very Low Birth Weight (VLBW) babies. The mothers were recruited only when their child’s life was no longer at risk; neonatologists and nurses on the ward indicated that only after the first 20 days following admission to the NICU (28 days if the baby is extremely preterm) do the vital signs of the newborn begin to stabilise and, therefore, do we see a decrease in risk to life. The decision behind administering the Q-sort in this time was motivated by the fact that, if the mother is concerned about her child’s life, the fear of death prevails over other emotions, preventing her from reflecting on herself and her way of parenting [75,76].

Before starting the first administration of the tool, all mothers were asked to provide informed consent to participate in the study, and for data processing.

Second administration (T2): 15/20 days after discharge, on the occasion of the first follow-up meeting at the Neonatal Clinic.

The research project started after having obtained the approval of the Ethical Committee of the Hospital.

### 2.5. Data Treatment and Statistical Analyses

The data, codified under the procedures set out in the reference test guide, were analysed using the Statistical Program for Social Sciences—SPSS (IBM SPSS Statistics for Windows, Version 27.0. IBM Corp., Armonk, NY, USA), through descriptive and parametric statistics.

A paired sample *t*-test was also calculated to verify the hypothesis of possible differences between the averages of maternal role competence indicators measured by the Q-sort at T1 and T2 (Goal 1).

A Multivariate Statistical Analysis (MANOVA) was then carried out on the data obtained at T1 and T2 to identify the possible effect of the independent variables considered (degree of prematurity of the child’s birth, sociocultural level, marital status, equal status, type of childbirth, nature of pregnancy) on the perception of maternal competence.

In addition, average scores of maternal competence factors detecting at T1 and T2, were compared, using the calculation of the paired sample *t*-test, with cut-offs that define the competence profile, obtained from the judges’ method during Q-sort validation

## 3. Results

The descriptive analysis of the sample’s characteristics (see Table 1) highlighted average age 32.5 years, SD 6.1. Depending on the degree of prematurity, defined using gestational age and birth weight, 60 mothers were identified as “mothers of VLBW infants”, with a gestational age ranging between the 32nd week and 28th week and weighing between 1500 and 1000 g; 44 were “mothers of LBW infants”, with a gestational age <34 weeks and birth weight <2500 g.; 75% of the mothers were married, 64% of a medium sociocultural level (education), most were primiparae (59%), 70% had caesarean delivery; and in 32% of cases, the childbirth was the result of an Assisted Reproductive Technology (ART) pathway.

**Figure 1:**
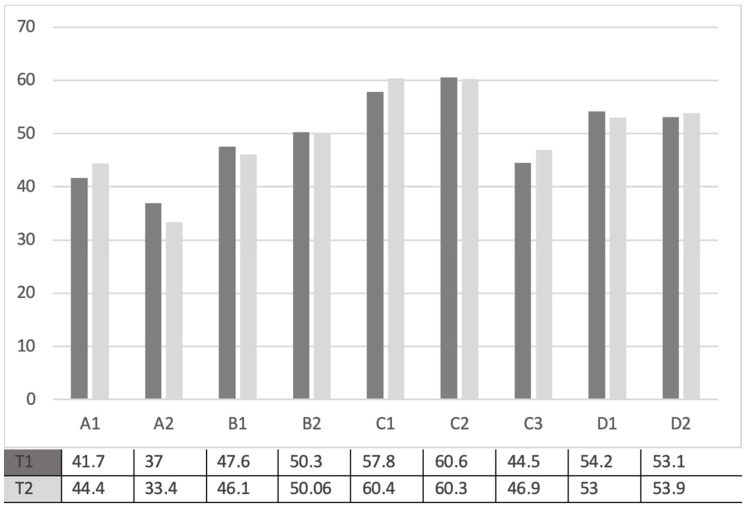
Configuration of the perceived maternal role competence in T1 and T2 (average indicator scores) (*n* = 104).

The distribution of average scores of maternal competence indicators showed, for both T1 and T2 timeframes (see Figure 1), a more consistent prevalence (higher averages) of the emotional and cognitive scaffolding indicators (C1 and C2); these mean scores were significantly higher than those of the competence profile, as per the paired sample *t*-test calculation (see Table 2 and Table 3).

In our sample, the highest averages beyond those of emotional and cognitive scaffolding were those relating to indicators of caregiving as responsiveness (D1) and flexible behavioural self-adjustment to the child’s needs (D2). In contrast, emotional coping ability (A1 and A2) appeared less distinctive. In addition, it was highlighted that both the caregiving and emotional coping scores were significantly lower than the competence criteria (see Table 2 and Table 3).

With regard to the comparison between the measurement of maternal competence perceived during the time of hospitalisation of the child in the NICU (T1) and then during the time of return home (T2) (see Table 4), the paired sample *t*-test showed: emotional coping, in terms of emotional self-regulation (A1), increased at T2 (*t* = 4.02; *p* = 0.001), however, in terms of decrease in emotional tension (A2) intended as the representation of one’s needs and search of a response to them (*t* = 2.7; *p* = 0.008), a decrease at T2 was found.

However, a statistically significant increase was found at T2 referring to emotional scaffolding (C1) as regards emotional availability and emotional monitoring (*t* = 3.35; *p* = 0.001), and to relational scaffolding (C3) (*t* = 2.43; *p* = 0.017). Furthermore, “caregiving” intended as responsiveness, emotional and cognitive attuning to the child’s needs (D1), significantly decreased at T2 (*t* = 4.27; *p* = 0.001). As for the other maternal role competence indicators, a strong tendency was found to maintain the same level from T1 to T2 (see Table 4).

Finally, the MANOVA data at T1 and T2 (see Table 5, Table 6, Table 7, Table 8, Table 9 and Table 10), highlighted many statistically significant differences among the maternal role competence indicators related to the different independent variables.

More specifically, considering the relationship ‘maternal role competence perception’ and the variable ‘child’s degree of birth prematurity’, measured at T1, cognitive (C2) (F = 17.9, *p* = 0.001) and relational (C3) (F = 12.6, *p* = 0.001) scaffolding were significantly lower in the mothers of very preterm babies. A greater degree of child prematurity seems to have had an effect at T1, even when compared with caregiving intended as the ability to be responsive and emotional and cognitively attuned to the child (D1), which was significantly lower in mothers of very preterm children (F = 6.2, *p* = 0.01), while cognitive coping (B1) appears significantly lower in mothers of moderately preterm children (F = 5.47, *p* = 0.02).

The variable “mother’s sociocultural level” showed significant differences at T1 with regard to caregiving intended as a flexible behavioural self-adjustment to the child’s needs (D2) (F = 3.12, *p* = 0.04), which appeared more present among mothers with a low or high sociocultural level. At T2, cognitive coping as regards the assessment of problems related to preterm birth (B2) appeared more present among mothers with a higher sociocultural level (F = 3.09, *p* = 0.05).

With regard to the effects of the variable “civil status”, statistically significant differences were found (only at T1) in relation to the following: emotional coping as regards self-regulation of one’s emotions (A1), which appeared higher in married mothers (F = 3.98, *p* = 0.049); cognitive coping as assessment of problems related to preterm birth (B2), which was also higher in married mothers (F = 7.48, *p* = 0.007); finally, emotional scaffolding as emotional availability (C1) (F = 0.37, *p* = 0.008), and cognitive scaffolding (C2) (F = 5.7, *p* = 0.01), which were more present among the mothers who cohabit with the child’s father than the married ones.

Considering the influence of the variable “type of childbirth”, significant differences were found only at T2 with regard to relational scaffolding (C3), the prevalence of which appeared significantly higher among mothers who had had a caesarean delivery (F = 3.6, *p* = 0.02).

The variable ‘parity’ only showed statistically significant differences at T1 with regard to emotional coping as self-regulation of one’s emotions (A1), which was higher among primiparae (F = 4.05, *p* = 0.04), and cognitive coping as the assessment of problems related to preterm birth (B2), which was also higher in primiparae (F = 4.5, *p* = 0.03).

Finally, only at T1, a significant difference was detected between mothers who had undergone assisted reproductive technology (ART) treatment and mothers who had had a natural pregnancy with regard to cognitive coping (B2), which appeared higher in mothers of babies born out of ART treatments (F = 10.8, *p* = 0.001).

## 4. Discussion

The data obtained through the Maternal Competence Q-sort highlighted that the mothers perceived themselves in negative terms compared to many areas of parental role competence. They showed low self-assessment, starting with the ability to parent their premature infant properly by attuning to the child’s special needs. Moreover, this caregiving function appeared to be even more failing after coming back home (T2). As evaluation of the differences between T1 and T2 showed, the mothers’ ability to be adequately responsive appears to be particularly complex, even when, on returning home, the risk had been overcome [77]. We could say that, paradoxically, the eagerly awaited homecoming takes the shape of a novel condition that is somewhat risky for maternal role competence.

In this regard, it is worth noting that frailty in the child’s early development, among the various possible evolutionary impairments, may lead to an alteration of the child’s cry. According to some studies in the field [78,79,80], premature infants, even when they are older than a corrected age of 43 weeks, activate a much more intense cry, regarding frequency, duration, and sound emitted (Hz) [78], than full-term infants. Therefore, the premature child’s prolonged and intense cry elicits an extremely active response in mothers. However, when they realise that they do not understand the real meaning of the child’s signals, which is an essential condition in order to feel confident in searching for solutions and making decisions, they may feel extremely distressed.

In other words, once at home, the preterm infant often displays higher behavioural instability and irritability because of the interruption to the hospital routine; this does not help parents who have not been adequately prepared; the mothers, often, express real feelings of failure as parents [36]. It would appear that the support of the physicians–nurses–psychologists team from the NICU plays an essential role in mediating between the special needs of the child and caring for the mother, helping her to feel responsive and aware of the events. At home, despite their best efforts, relatives cannot provide the mother with similar mediation and supportive functions, a fact which may induce new fears and challenges [66,81].

The difficulties that mothers face in this caregiving function leads to a weakening in their ability to give new meanings to challenges associated with caring for the child. This is coupled with having to attune adequately to the child’s special needs and to know how to recognise the child’s evolutionary characteristics and developmental stages in comparison with the corrected age. That means that they should always keep in mind their child’s birth weight in order to avoid making continuous comparisons with the evolutionary stages of full-term children. They should focus on the child’s evolutionary resources and not just on the most obvious impairments [82]. They need to be supported and accompanied, starting from the time of hospitalisation [66,83,84].

The aforementioned low self-esteem as properly responsive mothers, was also combined with self-devaluation related to the ability to recognise one’s emotional needs induced by special-evolutionary-need child [85].

Therefore, it would appear that the mothers involved in the study found it particularly challenging to step back from the negative emotions (fears, anxiety, doubts, etc.) ensuing from the risk condition [86,87].

Once again, on returning home, mothers do not appear to feel more confident in facing their negative emotional states, particularly when seeking help [35]. Probably, this difficulty in expressing, sharing, and recognising one’s emotional needs most likely calls into question the weight of behaviours of relatives, especially grandparents, but also brothers and friends, who tend to focus exclusively on their happiness in relation to the arrival of the child. They usually advise the mother to relax and enjoy her arrival at home with her child and family. The grandmothers, often, damage many contradictory advice, which can adversely affect the thoughts and emotions of mothers [81]; however, grandmothers are a point of reference for new mothers, especially in the first period after discharge from hospital, assuming not only the role of model to refer to, but also the concrete source of help and advice; therefore it is very important that even grandmothers are aware of the preterm birth specificity conditions and that they can better understand the concerns of new mothers.

Thus, as some studies suggest [81,88], grandmothers should be involved in the early intervention program in NICU and in returning home to support the management of maternal functions, such as breastfeeding.

In addition to self-devaluations such as these, however, the emotional, cognitive and relational scaffolding functions constitute an exception. In this regard, mothers appear to overestimate themselves and their self-confidence tends to increase at home. This attitude can be explained by the fact that, once at home, the closest family members (partners, parents, and siblings) typically offer mothers practical support.

This ambivalence in the mother, tending to vacillate between positive and negative misperceptions of different aspects of parenting competence, leads us to highlight the crisis of the maternal role competence in the child’s preterm birth conditions, which becomes a risk factor for the health of the child’s development [89]. As Pediatric Psychology suggests, the mother who demonstrates such unregulated behaviour ultimately becomes dysfunctional for the child’s life, conveying feelings of anxiety, restlessness, doubts, and hyper-control in the mother–child relationship [90].

Apart from these considerations, it is of interest to analyse the specific configuration of the perceived maternal role competence, during the two timeframes considered, in relation to the degree of prematurity of the child’s birth [20,91]. It should be pointed out that the general tendency of mothers to perceive themselves as not adequately equipped in the management of the parenting functions seems to be mainly induced by the degree of prematurity of the child, however, only during the time of hospitalisation in the NICU (T1) and only in relation to certain parenting functions only. The differences highlighted concerning the degree of prematurity are the functions of cognitive and relational scaffolding, and of caregiving as responsiveness and attunement to the child’s needs, which are more compromised in mothers of very premature babies. This result can be explained when we consider the fact that the greater fragility and reduced ability of the infant to react to external stimulations make it more difficult for the mother to attract her child’s attention, to understand how to recognise their needs and attune to those needs, to build a routine without the help of the medical staff [3,22,28,29,30].

Worth of note, however, is the perception of the parenting function cognitive coping, which appeared limited in the mothers of moderately preterm children. It can be assumed that, unlike the mothers of children at greater risk, they are less involved in cognitively reshaping the premature birth event maybe because they felt there to be less risk to life or, in any case, less risk to the development needs of the child.

The effects of the seriousness of the preterm birth do not appear significant in the management of the maternal role competence at home.

However, interesting to note is the weight of the variable “mother’s sociocultural level” compared with the perception of the maternal role competence, where a higher level, for example, positively influenced only the rational understanding of events linked to the child’s prematurity [92,93]. It would seem that the mother’s cultural level may ease cognitive coping because it provides her with more educational tools to reshape the attribution of meaning to events, and to understand the main difficulties that she is likely to face during her child’s first months of life; however, only after returning home.

As for the influence of other structural variables on the mothers’ self-perception as competent parents, the mothers who had followed medically assisted procreation treatment appear more confident in the cognitive coping function, that is, the ability to know how to assess the problems related to prematurity and hospitalisation as well as to recognise the resources to deal with them.

Women who undergo Assisted Reproductive Technology treatment have to face long-time-desired, complex, but also risky and uncertain births. Women whose pregnancy path has been almost totally guided tend to want to control everything when their real baby arrives and depends upon their care [94,95,96]. They, therefore, tend to evaluate each difficulty and look for a way to manage it; also, because ART treatment prepares couples for the possibility of premature birth, facilitating their cognitive coping ability.

### 4.1. Implications for Early Intervention Program to Support Maternal Competence

Therefore, based on the results of our study, we have defined an early psichoeducational intervention program, which is undergoing validation, to support and accompany maternal competence during the transition from hospital to home [31,45,97,98]. It is a process of change that evokes different types of needs: information needs on child development and possible strategies to take care of him, needs for social support, and needs related to parental role management with a premature baby.

The intervention program intends precisely to respond to these needs of mothers, and the use of Q-sort is a core. In fact, this technique can initiate in mothers a process of strengthening and/or redefining oneself as competent parents, creating “other” experiential spaces with them and starting, already in hospital, a process of self-remapping [10,99].

Our intervention program would be activated from the first weeks of the child’s hospitalisation in the NICU, through segments of parenting-competence guidance concerning mainly the recognition of the child’s expected evolutionary peculiarity which will be carefully monitored during follow-ups in the first 3 months of the child’s life.

The integrated work in the NICU between psychologists and nurses is considered fundamental; they can guide the mother in the caregiving skill, as recognition of child’s resources ability, and in the empowerment of self-image redefinition as a parent. As our study’s data suggest, related to the importance of relatives in the transition from hospital to home, the program also includes, in specific moments, the involvement of fathers and grandmothers, where present.

The program includes *n*. 4 steps and specifically:

- *1st Step (1st and 2nd week of hospitalisation of the child in NICU): “Contacting and get to know the child”*

In the hospital rooms, the psychologist and the nurse together, in front of the children’s cradles, provide mothers with information on the developmental characteristics usually present in premature babies, answering any questions from mothers.

A booklet entitled “My child and our relationship” is then given to each mother, a sort of diary to be filled in in the various steps of the program, to reflect together with the operators on herself as the mother of a premature child [97].

The use of this booklet allows the observation of the child in the cradle of the NICU in the absence of stimulation, the observation of the behavioural reactions of the child during specific stimulations (mother’s voice and touch), and finally allows the mother to focus on perception of themselves as a parent with that specific child [100].

In this step, there is an intervention by the psychologist who also involves the father in front of the cradle: starting from the observations made by mother thanks to the booklet, the comparison between the two parents is stimulated on the child’s resources that they are able to share, and on the frailties that they worry most. The psychologist activates counselling segments with the parental couple and the nurse deals with educational segments on child care; all aimed at strengthening the cohesion of the couple in managing the child’s care and making them both feel a little more competent.

Our intervention program also includes a “laboratory of contact” with the child that allows the mother to be supported in implementing contact behaviours with the child first in the presence of psychologist, and after alone. Furthermore, a nurse expert in kangaroo therapy activates with mothers an experience of skin-to-skin mother–child contact

The experience of “skin-to-skin care” can improve the infant’s sleep time and temperature regulation, decreased crying and need for oxygen, increases parental confidence and positive infant–parent interaction [101,102,103]. The experience of “skin-to-skin care” may help parents to be acquainted with their infant and thus prepare for the transition to home.

These contact activities will be repeated by the mothers every day. At the end of each contact experience, the mother is encouraged by the booklet to reflect on how she felt, and she is also asked to share the experience lived with the child with her partner.

- *2nd Step (between the 3rd and 4th week of hospitalisation of the child): “Talking to the child”*

The psychologist activates with the mother, in front of her baby’s cradle, a narration laboratory, explaining the importance of reading for the development of premature babies, in particular for brain development.

Thanks to this laboratory, the mother chooses a fairy tale to read to her child [104].; finishing the narration, the observations in the booklet will lead her to recognise the reactions of her own child on hearing her voice.

This involvement will enable mothers to recognise their child’s evolutionary individualities and changes, and to acquire operational strategies to support their child’s development and take care of his/her special evolutionary needs [83].

- *3rd Step: (between 4th and 6th weeks of hospitalisation): “Look at Yourself as Mothers and Talk to Own Mother”*

In this 3rd Step, some information is provided for mothers and grandmothers about how to care for premature babies at home without the support of the NICU staff.

In this way, grandmothers will be more aware of the preterm birth condition and the care required and will be able, once home, to be more able to connect with the possible difficulties of the baby’s mother.

At the same time, the Q-sort on perceived maternal competence is administrated for each mother [105].

From the moment of application of the Q-sort until discharge, the psychologist supports mothers in the redefinition of maternal competence, through individual counselling interventions [106,107].

- *4th Step: “The Return Home”*

During the first month after discharge, the psychologist and the nurse separately carry out an interview (telephone or online video call) with a follow-up function and offer of informative and/or emotional support [108].

At the follow-up visit scheduled for the 1st month after discharge, the psychologist administers the Q-sort to the mother again and sets an appointment for the return of the data; during this online meeting, starting from a comparison between the q-sort data during hospitalisation and the one completed one month after discharge, the psychologist activates the counselling, underlining the changes and supporting any further changes that appear necessary for the child’s well-being and the relationship with the child [109].

We are currently verifying the effectiveness of this psychoeducational intervention program to support the transition from hospital to home; in fact, it has been hypothesised that it may have a direct effect of reducing maternal distress (anxiety, parenting stress, depressive symptoms, worry about child health, child’s fragilities perception), and above all of enhancing the perceived sense of parental competence, in particular in respect to the caregiving function.

Therefore, it is hypothesised that the well-being of the mother can have a positive effect on the child’s development (adequate achievement of developmental goals according to the correct age, on a cognitive, motor, social, emotional level) and on the quality of the mother–child relationship, in terms of sensitivity, responsiveness and reciprocity [9,53,54,92,110].

These effects are monitored from the time of hospitalisation in NICU up to the 3rd month of the correct age of the child, making use of the comparison with a control group consisting of a group of mothers with the same sociodemographic characteristics but subjected to routine care of the ward.

### 4.2. Strengths and Limits

The strengths of the study include the longitudinal perspective, as the measurement of maternal competence perceived both during hospitalisation and at discharge, a perspective not frequently found in literature. Its strength lies also in the Q-methodology-based research tool adopted. It is a specific self-observation tool that allows mothers to immediately face their self-image as preterm-baby parents. By stimulating reflections of themselves and their mother–child relationship, it might lead to behavioural change in the mother [10].

Therefore, the Q-sort tool, while allowing assessment, also activates an intervention functional to the change process. It is, thus, a tool built specifically for use with mothers of preterm infants, while research often uses tools adapted for use with parents of premature infants.

Another strength consists in the fact that the data collected from the administration of the Q-sort allowed the planning and implementation of clinical interventions. Finally, a further strength is the type of variable that is addressed, that is, parental competence as a complex construct, while other studies have focused above all on anxiety, depression and parental stress levels and only in only some cases the relationship with the child but never parental competence [92].

The shortcomings of the study consist in the small number of the sample; however, it is a sample characterised by a specific risk condition and, therefore, it is difficult to recruit larger numbers.

The most important strength consists in the fact that the data collected from the administration of the Q-sort allowed the planning and implementation of early intervention program to support the mothers in the transition from hospital to home. However, this program can only partially involve fathers because a Fathers Q-sort Version is not available; we are working on the production of this Q-sort version. This means the possibility to prepare mothers and fathers together with adequate management of the transition along the path of awareness, empowerment and change, activated by the tool. The tool can become a strategy for identifying the needs of fathers, but also to encourage them to make a contribution in facilitating the family’s adaptation to the new condition.

### 4.3. Conclusions

The study made it possible to have data on a construct that is almost not investigated in preterm birth conditions: the parental competence [10,11], in particular that perceived by mothers. It is sufficient to say that the Q-sort is the only tool in Italian, currently available specifically for mothers of preterm babies.

The focus of this construct goes beyond the classic consideration of parents’ psychological condition, because there is always an eye towards the child, the relationship with him and the support for his development.

From this perspective, the data made us discover that the mothers of preterm babies experience greater difficulties as parents than a responsiveness to the child, intended as ability to recognise his special needs, linked to his prematurity; we found that this perception of incompetence can persist or even increase on returning home, when the constant support of the health team is lacking.

Furthermore, we discovered that there is another important dimension of maternal competence in crisis especially after discharge, namely emotional coping, in the sense of the ability to respond to one’s emotional needs related to concerns for one’s child; this data suggests the presence of emotional stress, certainly not functional to a good management of parental functions.

Speaking of parental competence means, in other words, referring to what can constitute the greatest external resource for promoting the development of a child who comes into the world in a condition of fragility.

For this reason, in the light of the data, we wanted to create a specific program to support maternal competence, which while focusing primarily on mother, considers the involvement of father as well as grandmothers, in some topical moments.

## Figures and Tables

**Table 1:** Characteristics of the sample (*n* = 104).

Mothers’ Characteristics (*n* = 104)	
Age in years, mean (SD)	32.5 (SD 6.1)
Prematurity of children	
VLBW	60 (58%)
LBW	44 (42%)
Education level	
High	12 (11%)
Medium	66 (64%)
Low	26 (25%)
Marital status	
Married	78 (75%)
Live-in partners	26 (25%)
Parity	
Primiparae	61 (59%)
Multiparae	43 (41%)
Type of delivery	
Caesarean section	74 (71%)
Assisted Reproductive Technology	33 (32%)

**Table 2:** Comparison between the Q-sort scores obtained by the sample and the maternal role competence criteria at T1 (paired sample *t*-test) (*n* = 104).

Maternal Role Competence Indicators		Mean	Differences between Means	T (df = 103)	*p* Value
A1	Sample	4.2	−0.38	−6.26	0.001 *
	Judges	4.5			
A2	Sample	3.7	−0.79	−9.53	0.001 *
	Judges	4.5			
B1	Sample	4.7	0.08	−1.14	0.25
	Judges	4.7			
B2	Sample	5	−0.37	−6.74	0.001 *
	Judges	5.4			
C1	Sample	5.7	0.98	16.24	0.001 *
	Judges	4.8			
C2	Sample	6	1.33	22.96	0.001 *
	Judges	4.7			
C3	Sample	4.4	−0.21	−3.16	0.002 *
	Judges	4.7			
D1	Sample	5.7	−0.19	−3.22	0.002 *
	Judges	5.9			
D2	Sample	5.3	−0.28	−4.33	0.001 *
	Judges	5.6			

A1—emotional self-regulation; A2—decrease in emotional tension; B1—cognitive reshaping of the preterm birth event; B2—assessment of the problems related to preterm birth and hospitalisation in NICU; C1—emotional scaffolding; C2—cognitive scaffolding; C3—relational scaffolding; D1—responsiveness; D2—flexible behavioural self-adjustment to the child’s needs. Significant results are marked with *.

**Table 3:** Comparison between the Q-sort scores obtained by the sample and the maternal role competence criteria at T2 (paired sample *t*-test) (*n* = 104).

Maternal Role Competence Indicators		Mean	Differences between Means	T (df = 103)	*p* Value
A1	Sample	4.5	−0.10	−1.53	0.12
	Judges	4.5			
A2	Sample	3.4	−1.10	−14.25	0.001 *
	Judges	4.5			
B1	Sample	4.7	−0.072	−1.26	0.20
	Judges	4.7			
B2	Sample	5	−0.42	−7.9	0.001 *
	Judges	5.4			
C1	Sample	6	1.25	18.18	0.001 *
	Judges	4.8			
C2	Sample	5.9	1.20	18.78	0.001 *
	Judges	4.7			
C3	Sample	4.7	−0.013	−0.19	0.84
	Judges	4.7			
D1	Sample	5.4	−0.49	−10.1	0.001 *
	Judges	5.9			
D2	Sample	5.3	−0.25	−5.71	0.001 *
	Judges	5.6			

A1—emotional self-regulation; A2—decrease in emotional tension; B1—cognitive reshaping of the preterm birth event; B2—assessment of the problems related to preterm birth and hospitalisation in NICU; C1—emotional scaffolding; C2—cognitive scaffolding; C3—relational scaffolding; D1—responsiveness; D2—flexible behavioural self-adjustment to the child’s needs. Significant results are marked with *.

**Table 4:** Factors of the Q-Sort and differences between T1 and T2 (paired sample *t*-test) (*n* = 104).

Comparison of the Q-Sort Factors between the Time (T1 and T2)		Mean	Difference between Means	T (df = 103)	*p* Value
A1	T1	41.9	−2.7	−4.02	0.001 *
	T2	44.7			
A2	T1	37	3.07	2.7	0.008 *
	T2	34			
B1	T1	46.7	−0.09	−0.13	0.89
	T2	46.7			
B2	T1	50.5	0.57	0.78	0.43
	T2	49.9			
C1	T1	57.8	−2.70	−3.35	0.001 *
	T2	60.6			
C2	T1	60.8	1.22	1.41	0.16
	T2	59.6			
C3	T1	44.8	−2.01	−2.43	0.017 *
	T2	46.8			
D1	T1	57	2.97	4.27	0.001 *
	T2	54			
D2	T1	53.2	−0.17	−0.24	0.81
	T2	53.4			

A1—emotional self-regulation; A2—decrease in emotional tension; B1—cognitive reshaping of the preterm birth event; C1—emotional scaffolding; C2—cognitive scaffolding; C3—relational scaffolding; D1—responsiveness; D2—flexible behavioural self-adjustment to the child’s needs. B2—assessment of the problems related to preterm birth and hospitalisation in NICU. Significant results are marked with *.

**Table 5:** Maternal competence (Q-sort scores) in NICU (T1) in relation to levels of severity of preterm birth, mother’s sociocultural level (MANOVA) (*n* = 104).

Q-Sort Indicators	*Levels of Severity of Preterm Birth*		*Mother’s Sociocultural Levels*		
	VLBW	LBW	Low	Medium	High
	(*n* = 60)	(*n* = 44)	(*n* = 26)	(*n* = 66)	(*n* = 12)
	*M* (*SD*)		*M* (*SD*)		
A1	42.7	40.8	42	42	41
	−5.8	−6.4	−5.6	−6.6	−3
	F = 2.6 *p* = 0.10		F = 1.05 *p* = 0.90		
A2	37.8	36	35.8	37.7	36
	−9.9	−5.8	−6.9	−9.4	−4.9
	F = 1.23 *p* = 0.26		F = 0.55 *p* = 0.57		
B1	48	44	45.6	46.7	48.6
	−6.6	−7.7	−7.7	−7.6	−2.7
	F = 5.47 *p* = 0.02 *		F = 0.72 *p* = 0.48		
B2	51.2	49.24	49.2	50.8	51.2
	−5.8	−5.1	−5.8	−5.4	−5.8
	F = 2.6 *p* = 0.10		F = 0.84 *p* = 0.43		
C1	58.1	57.5	59	57.4	57.8
	−6.6	−5.6	−5.7	−6.3	−6.9
	F = 0.21 *p* = 0.64		F = 0.67 *p* = 0.51		
C2	58.8	63.4	61.4	61	58
	−5.3	−5.7	−6	−5.7	−6.2
	F = 17.9 *p* = 0.001 *		F = 1.59 *p* = 0.20		
C3	42.8	47.5	45	45	43.2
	−7.5	−4.8	−5	−7.7	−5.6
	F = 12.6 *p* = 0.001 *		F = 0.35 *p* = 0.70		
D1	55.7	58.7	56	57	58.1
	−6.1	−5.8	−6.6	−6.3	−4
	F = 6.2 *p* = 0.01 *		F = 0.36 *p* = 0.69		
D2	54.2	51.8	55.3	52	55.1
	−6.8	−5.8	−4.9	−7.2	−2.8
	F = 3.4 *p* = 0.06		F = 3.12 *p* = 0.04 *		

**Note:** maternal competence indicators. A1—emotional self-regulation; A2—decrease in emotional tension; B1—cognitive reshaping of the preterm birth event; B2—assessment of the problems related of preterm birth and hospitalisation in NICU; C1—emotional scaffolding; C2—cognitive scaffolding; C3—relational scaffolding; D1—responsiveness; D2—flexible behavioural self-adjustment to the child’s needs. Significant results are marked with *.

**Table 6:** Maternal competence (Q-sort scores) in NICU (T1) in relation to type of delivery and parity (MANOVA) (*n* = 104).

Q-Sort Indicators	*Type of Delivery*		*Parity*	
	Natural Childbirth	Caesarean Section	Primiparous	Multiparous
	(*n* = 29)	(*n* = 75)	(*n* = 61)	(*n* = 43)
	*M* (*SD*)		*M* (*SD*)	
A1	43	41.6	42.9	40
	−6.5	−5.9	−5.6	−6.5
	F = 1.2 *p* = 0.29		F = 4.05 *p* = 0.04 *	
A2	38.3	36.5	37.1	36.9
	−9.8	−7.9	−8.4	−8.6
	F = 0.72 *p* = 0.48		F = 0.02 *p* = 0.88	
B1	47.6	46.5	46.9	46.3
	−9.2	−6.3	−7.5	−6.9
	F = 1.5 *p* = 0.21		F = 0.19 *p* = 0.66	
B2	48.7	51.2	51.4	49.1
	−5.5	−5.5	−5.5	−5.5
	F = 2.4 *p* = 0.08		F = 4.5 *p* = 0.03 *	
C1	57.8	57.8	57.8	57.9
	−7.7	−5.6	−6.7	−5.4
	F = 0.05 *p* = 0.94		F = 0.02 *p* = 0.88	
C2	59.8	61	60	61.9
	−5.6	−5.9	−5.7	−6
	F = 2.2 *p* = 0.10		F = 2.6 *p* = 0.10	
C3	43.4	45.2	43.8	46.2
	−8.1	−6.3	−6.7	−7
	F = 1.8 *p* = 0.17		F = 2.9 *p* = 0.08	
D1	56.9	57	56.4	57.7
	−5.4	−6.5	−6	−6.4
	F = 0.003 *p* = 0.99		F = 1.19 *p* = 0.27	
D2	53.3	53.4	53.1	53.3
	−3.8	−4.9	−6.2	−6.9
	F = 1.01 *p* = 0.36		F = 0.01 *p* = 0.90	

**Note:** Maternal competence indicators. A1—emotional self-regulation; A2—decrease in emotional tension; B1—cognitive reshaping of the preterm birth event; B2—assessment of the problems related of preterm birth and hospitalisation in NICU; C1—emotional scaffolding; C2—cognitive scaffolding; C3—relational scaffolding; D1—responsiveness; D2—flexible behavioural self-adjustment to the child’s needs. Significant results are marked with *.

**Table 7:** Maternal competence (Q-sort scores) in NICU (T1) in relation to levels of marital status of the mothers and medically assisted procreation (MANOVA) (*n* = 104).

Q-Sort Indicators	*Marital Status*		*Assisted Reproductive Technology*	
	Married	Co-Habitant	Yes	No
	(*n* = 78)	(*n* = 26)	(*n* = 33)	(*n* = 71)
	*M* (*SD*)		*M* (*SD*)	
A1	42.6	39.8	42.9	41.4
	−6	−6.1	−4.6	−6.6
	F = 3.98 *p* = 0.049 *		F = 1.3 *p* = 0.25	
A2	36.9	37.3	36.4	37.3
	−8.9	−7	−6.3	−9.3
	F = 0.03 *p* = 0.85		F = 0.25 *p* = 0.61	
B1	47	45	45.8	47
	−6.7	−8.7	−5.7	−7.8
	F = 1.53 *p* = 0.21		F = 0.67 *p* = 0.41	
B2	51.3	47.9	53	49.3
	−5.2	−5.9	−5.5	−5.2
	F = 7.48 *p* = 0.007 *		F = 10.8 *p* = 0.001 *	
C1	57.6	58.8	57.2	58.1
	−6.4	−5.3	−5.6	−6.4
	F = 0.37 *p* = 0.008		F = 0.55 *p* = 0.46	
C2	60	63.1	60.2	61
	−5.4	−6.7	−5.6	−6
	F = 5.7 *p* = 0.01 *		F = 0.50 *p* = 0.47	
C3	44.1	47	45	44.7
	−7.1	−6	−5	−7.6
	F = 3.41 *p* = 0.06		F = 0.02 *p* = 0.87	
D1	56.8	57.3	56.4	57.2
	−6.1	−6.3	−6.6	−6
	F = 0.10 *p* = 0.74		F = 0.37 *p* = 0.54	
D2	53.1	53.6	52.4	53.5
	−6.9	−5.1	−4.7	−7.1
	F = 0.12 *p* = 0.73		F = 0.68 *p* = 0.41	

**Note:** Maternal competence indicators. A1—emotional self-regulation; A2—decrease in emotional tension; B1—cognitive reshaping of the preterm birth event; B2—assessment of the problems related of preterm birth and hospitalisation in NICU; C1—emotional scaffolding; C2—cognitive scaffolding; C3—relational scaffolding; D1—responsiveness; D2—flexible behavioural self-adjustment to the child’s needs. Significant results are marked with *.

**Table 8:** Maternal competence (Q-sort scores) at the time of home (T2) in relation to levels of severity of preterm birth and mother’s sociocultural level (MANOVA) (*n* = 104).

Q-Sort Indicators	*Levels of Severity of Preterm Birth*		*Mother’s Sociocultural Levels*		
	VLBW	LBW	Low	Medium	High
	(*n*= 60)	(*n* = 44)	(*n* = 26)	(*n* = 66)	(*n* = 12)
	*M* (*SD*)		*M* (*SD*)		
A1	44.9	44.4	45.3	44.3	45.5
	−6.6	−6.8	−5.3	−6.8	−8.7
	F = 0.13 *p* = 0.71		F = 0.30 *p* = 0.73		
A2	33.8	34.2	34.4	34.5	30
	−8.1	−7.5	−6.9	−7.3	−11.5
	F = 0.05 *p* = 0.82		F = 1.7 *p* = 0.17		
B1	47.5	45.7	45.4	46.8	49.1
	−6.4	−4.6	−4.7	−6.4	−3.3
	F = 2.2 *p* = 0.13		F = 1.76 *p* = 0.17		
B2	49.6	50.3	47.8	50.2	52.2
	−5.8	−5.1	−5.6	−5.5	−3.9
	F = 0.45 *p* = 0.50		F = 3.09 *p* = 0.05 *		
C1	60.8	60.2	62.7	60.2	58
	−7	−7	−5.9	−7.2	−8
	F = 0.21 *p* = 0.64		F = 2.05 *p* = 0.13		
C2	59.5	59.7	61.6	59	58.4
	−6.3	−6.9	−5.9	−7.2	−2.2
	F = 0.02 *p* = 0.88		F = 1.64 *p* = 0.19		
C3	46.2	47.7	46.7	47	46.2
	−6.2	−8.1	−4.9	−7.3	−10.1
	F = 1 *p* = 0.32		F = 0.06 *p* = 0.93		
D1	53.6	54.6	52.7	54.2	55.8
	−5.4	−4.4	−6.2	−4.4	−5.3
	F = 1.03 *p* = 0.31		F = 1.65 *p* = 0.19		
D2	53.7	52.9	52.9	53.3	54.7
	−4.8	−4.3	−4.5	−4.5	−5.6
	F = 0.71 *p* = 0.040		F = 0.64 *p* = 0.52		

**Note:** Maternal competence indicators. A1—emotional self-regulation; A2—decrease in emotional tension; B1—cognitive reshaping of the preterm birth event; B2—assessment of the problems related of preterm birth and hospitalisation in NICU; C1—emotional scaffolding; C2—cognitive scaffolding; C3—relational scaffolding; D1—responsiveness; D2—flexible behavioural self-adjustment to the child’s needs. Significant results are marked with *.

**Table 9:** Maternal competence (Q-sort scores) at the time of home (T2) in relation to type of delivery and parity (MANOVA) (*n* = 104).

Q-Sort Indicators	*Type of Delivery*		*Parity*	
	Natural Childbirth	Caesarean Section	Primiparous	Multiparous
	(*n* = 29)	(*n* = 75)	(*n* = 61)	(*n* = 43)
	*M* (*SD*)		*M* (*SD*)	
A1	45.6	44.3	45	44.2
	−8.8	−5.8	−6.6	−6.7
	F = 0.40 *p* = 0.67		F = 0.38 *p* = 0.53	
A2	35.6	33.4	34.7	32.8
	−7.9	−7.8	−7.4	−8.4
	F = 1.21 *p* = 9.30		F = 1.48 *p* = 0.22	
B1	47.8	46.4	47.2	46
	−7.4	−5	−6.5	−4.5
	F = 1.10 *p* = 0.33		F = 1.02 *p* = 0.31	
B2	50.1	49.9	49	51.1
	−5.9	−5.4	−5.3	−5.6
	F = 0.2 *p* = 0.76		F = 3.5 *p* = 0.06	
C1	59.9	60.8	60.3	60.8
	−8.8	−6.3	−7.7	−6
	F = 0.37 *p* = 0.68		F = 0.13 *p* = 0.71	
C2	59.7	59.4	659.8	59.1
	−6.2	−6.6	−6.5	−6.6
	F = 1.29 *p* = 0.27		F = 0.28 *p* = 0.59	
C3	43.9	47.8	46.2	47.7
	−8.5	−6.3	−7.5	−6.5
	F = 3.6 *p* = 0.02 *		F = 1.1 *p* = 0.28	
D1	53.7	54.1	54.2	53.7
	−5.3	−4.9	−4.9	−5.1
	F = 0.15 *p* = 0.85		F = 0.19 *p* = 0.65	
D2	53.2	53.2	53	53.8
	−3.8	−4.9	−4.3	−5
	F = 0.49 *p* = 0.61		F = 0.78 *p* = 0.37	

**Note:** Maternal competence indicators. A1—emotional self-regulation; A2—decrease in emotional tension; B1—cognitive reshaping of the preterm birth event; B2—assessment of the problems related of preterm birth and hospitalisation in NICU; C1—emotional scaffolding; C2—cognitive scaffolding; C3—relational scaffolding; D1—responsiveness; D2—flexible behavioural self-adjustment to the child’s needs. Significant results are marked with *.

**Table 10:** Maternal competence (Q-sort scores) at the time of home (T2) in relation to marital status and medically assisted procreation (MANOVA) (*n* = 104).

Q-Sort Indicators	*Marital Status*		*Assisted Reproductive Technology*	
	Married	Yes	No	Co-Habitant
	(*n* = 78)	(*n* = 33)	(*n* = 71)	(*n* = 26)
	*M* (*SD*)		*M* (*SD*)	
A1	45	44.6	44.6	43.5
	−6.9	−4.6	−7.4	−5.9
	F = 1.03 *p* = 0.31		F = 0.000 *p* = 0.99	
A2	33.9	34.7	33.7	34.3
	−8.3	−6.8	−8.3	−6.5
	F = 0.05 *p* = 0.81		F = 0.37 *p* = 0.54	
B1	47.2	46.9	46.7	45.4
	−6	−5.7	−5.8	−4.7
	F = 2.02 *p* = 0.15		F = 0.05 *p* = 0.82	
B2	50	49.3	50.2	49.5
	−5.4	−4.1	−6	−5.8
	F = 0.19 *p* = 0.66		F = 0.47 *p* = 0.49	
C1	60	60.2	60.7	62
	−6.9	−7.3	−6.9	−7.3
	F = 1.6 *p* = 0.20		F = 0.10 *p* = 0.74	
C2	59.1	59.1	59.8	61
	−6.2	−6	−6.8	−7.5
	F = 1.77 *p* = 0.18		F = 0.22 *p* = 0.064	
C3	46.6	47.4	46.6	47.6
	−7.6	−6.5	−7.4	−5.3
	F = 0.42 *p* = 0.51		F = 0.29 *p* = 0.58	
D1	54.5	54	54	52.7
	−4.4	−4.6	−5.2	−6.3
	F= 2.34 *p*= 0.12		F = 0.000 *p* = 0.99	
D2	53.4	53.3	53.4	53.3
	−4.5	−4	−4.9	−4.9
	F = 0.005 *p* = 0.94		F = 0.004 *p* = 0.95	

**Note:** Maternal competence indicators. A1—emotional self-regulation; A2—decrease in emotional tension; B1—cognitive reshaping of the preterm birth event; B2—assessment of the problems related of preterm birth and hospitalisation in NICU; C1—emotional scaffolding; C2—cognitive scaffolding; C3—relational scaffolding; D1—responsiveness; D2—flexible behavioural self-adjustment to the child’s needs.

## Data Availability

The data presented in this study are available on request from the corresponding author.
